# An Orientation Dependent Size Illusion Is Underpinned by Processing in the Extrastriate Visual Area, LO1

**DOI:** 10.1177/2041669516667628

**Published:** 2016-09-26

**Authors:** Kyriaki Mikellidou, André D. Gouws, Hannah Clawson, Peter Thompson, Antony B. Morland, Bruce D. Keefe

**Affiliations:** Department of Psychology, University of York, UK; University of Pisa, Italy; York Neuroimaging Centre, Department of Psychology, University of York, UK; Department of Psychology, University of York, UK; Department of Psychology, University of York, UK; York Neuroimaging Centre, Department of Psychology, University of York, UK; Centre for Neuroscience, Hull-York Medical School, UK; York Neuroimaging Centre, Department of Psychology, University of York, UK

**Keywords:** fMRI, perception, spatial vision, TMS

## Abstract

We use the simple, but prominent Helmholtz’s squares illusion in which a vertically striped square appears wider than a horizontally striped square of identical physical dimensions to determine whether functional magnetic resonance imaging (fMRI) BOLD responses in V1 underpin illusions of size. We report that these simple stimuli which differ in only one parameter, orientation, to which V1 neurons are highly selective elicited activity in V1 that followed their physical, not perceived size. To further probe the role of V1 in the illusion and investigate plausible extrastriate visual areas responsible for eliciting the Helmholtz squares illusion, we performed a follow-up transcranial magnetic stimulation (TMS) experiment in which we compared perceptual judgments about the aspect ratio of perceptually identical Helmholtz squares when no TMS was applied against selective stimulation of V1, LO1, or LO2. In agreement with fMRI results, we report that TMS of area V1 does not compromise the strength of the illusion. Only stimulation of area LO1, and not LO2, compromised significantly the strength of the illusion, consistent with previous research that LO1 plays a role in the processing of orientation information. These results demonstrate the involvement of a specific extrastriate area in an illusory percept of size.

## Introduction

Recent studies have shown a link between perceived size and the spatial extent of BOLD responses in the primary visual cortex (V1) by presenting stimuli in the context of strong 2D depth cues (Fang, Boyaci, Kersten, & Murray, 2008; He, Mo, Wang, & Fang, 2015; Murray, Boyaci, & Kersten, 2006) or by manipulating the extraocular cues of vergence and accommodation (Sperandio, Chouinard, & Goodale, 2012). Perceived size and size constancy are thought to be computed beyond V1 (Blakemore, Garner, & Sweet, 1972) and this has led to the idea that feedback from higher visual areas modulates BOLD responses in V1, where both retinal and extraretinal signals must be brought together (Sterzer & Rees, 2006). Whether any perceptual illusion arises solely from V1-specific mechanisms (Michel, Chen, Geisler, & Seidemann, 2013; Pooresmaeili, Arrighi, Biagi, & Morrone, 2013) regardless of feedback projections from higher areas (Fang et al., 2008; Kok, Bains, van Mourik, Norris, & de Lange, 2016; Kok & De Lange, 2014; Murray et al., 2006; Sperandio et al., 2012), is an issue yet to be resolved (see Discussion section).

**Figure 1.**
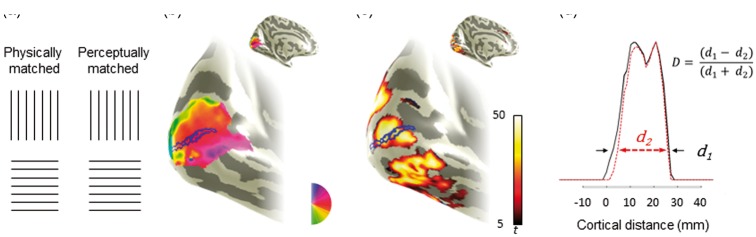
The Helmholtz squares illusion and the BOLD responses it induces in primary visual cortex. (a) On the left, a vertically lined square appears above a physically matched, horizontally lined square—note the lower square appears narrower than the upper square as first described by Helmholtz (Helmholtz & Southall, 1925). On the right, the horizontally lined stimulus has been widened so it is no longer physically square, but now appears to match the dimensions of the vertically lined square above it. The width of the perceptually matched horizontally lined stimulus was increased (by 8–18%) according to each individual’s psychophysics results. (b) BOLD response phase to rotating checkerboard wedges superimposed in false color on an inflated representation of the occipital lobe. Data have been restricted to V1. BOLD responses to squares and rectangles in (a) were analyzed over a line ROI, illustrated as a dark blue outline. This ROI extended along the representation of the horizontal meridian, where the inner and outer stimulus edges are represented. (c) The response (*t*-statistic) to all stimuli compared against a uniform gray background. (d) The BOLD response profile (*t*-statistic) as a function of cortical distance along the ROI, with negative *t*-values set to zero. The measures, *d*, for different stimulus conditions were computed by taking the width of the function at 25% of its peak value and were used to compute a contrast measure D for each hemisphere. *Note*. ROI = region of interest.

In this study, we tested whether processing specific to V1 can be linked to perceptual experience using the well-known Helmholtz squares illusion (Helmholtz & Southall, 1925; Figure 1a), in which a vertically striped square appears wider than a horizontally striped one of identical dimensions (Helmholtz & Southall, 1925). This illusion has the advantage of minimizing high-level feedback to V1 as it does not depend on misapplied cues for size-constancy scaling (Murray et al., 2006; Sperandio et al., 2012) and is therefore a good candidate to determine whether stimulus-driven BOLD responses in V1 alone underlies perceived size.

As others have (Kok et al., 2016; Kok & De Lange, 2014; Murray et al., 2006; Pooresmaeili et al., 2013; Sperandio et al., 2012), we measured the extent of BOLD responses in primary visual cortex, but in our case for horizontally and vertically lined rectangles, which elicit the Helmholtz illusion. We found that the extent of BOLD responses in primary visual cortex followed the physical, but not perceptual dimensions of the stimuli. However, our participants performed a demanding central fixation task during presentation of the stimuli, so attention was not on the stimuli. Consequently, we bias the BOLD responses that we will measure to stimulus-driven activity by reducing the effect of top-down feedback (Fang et al., 2008). We find no illusion-related BOLD responses in V1 consistent with the idea that stimulus-driven activity in V1 cannot solely explain the illusion (Fang et al., 2008; see Discussion). We next investigated whether activity in V1 and two candidate extrastriate visual areas (LO1 and LO2) played a causal role in the illusion. To do this, we applied transcranial magnetic stimulation (TMS) to one of the three cortical regions, while participants judged the aspect ratio of horizontally and vertically lined rectangles. The stimuli were set such that for each individual, they were normally perceived as square, despite being physically rectangular. We predicted therefore that TMS of a cortical site underpinning the illusion would generate a *release* from the illusion. Importantly, this release would register as an increase in the number of responses that correctly identify the physical aspect ratio of the stimuli that were normally perceived as perceptually square. Our results showed that TMS to LO1 increased the number of correct responses relative to TMS to other sites (LO2 and V1) and a no TMS control condition. This shows that even when a stimulus-related task is performed, which could arguably result in illusion-related signals being feedback to V1, the activity in V1 alone does not cause the illusion. We show, therefore, that extrastriate processing underpins this example of a size illusion.

## Materials and Methods

### Participants

Eight participants (six females; age range: 18–25 years old; mean = 21) carried out the pre-functional magnetic resonance imaging (fMRI) psychophysics experiment, retinotopic-mapping sessions, and the Helmholtz’s squares fMRI experiment. Seven additional participants carried out the pre-TMS psychophysics and TMS sessions (one female; age range 23–47; mean = 30). All participants had normal or corrected-to-normal visual acuity and no history of neurological impairments. Informed consent was obtained in accordance with the Declaration of Helsinki. Procedures and protocols were approved by the York Neuroimaging Centre (YNiC) Research Ethics Committee at The University of York, United Kingdom.

### Design of the fMRI Experiment

Our first approach to understand what neural processes contribute to the Helmholtz square illusion was to measure the extent of BOLD responses in V1 elicited by stimuli *inducing* and also *cancelling* the illusion. The logic is as follows: squares comprising vertical lines appear wider than squares comprising horizontal lines (Figure 1a), so BOLD responses along the representation of the horizontal meridian in V1 (Figure 1b and c) would be of greater extent for vertically than horizontally lined squares (e.g. black vs. red line in Figure 1d), if V1 activity follows the illusion. If the BOLD responses in V1 map the physical dimensions of the stimuli, however, no difference in the extent of the activity would register. If the horizontal lines are extended, such that the participant perceives the vertically and horizontally lined stimuli to be equal in width (see “perceptually matched” in Figure 1a), the extent of BOLD responses in V1 would be equal, if that activity follows the illusion. In contrast, the extent of V1 BOLD responses along the representation of the horizontal meridian would be greater for the horizontally lined than for the vertically lined stimulus, if V1 follows the physical dimension of the stimulus.

In terms of the fMRI design, we presented four stimulus conditions. In general, horizontally lined stimuli presented at an eccentricity of 3° were paired with vertically lined squares presented at an equal eccentricity in the opposite hemifield. We varied the hemifield in which the horizontally lined stimulus appeared (left or right) and the width of the horizontally lined stimulus (physically or perceptually square). As a result, two of the stimulus conditions comprised the vertically lined squares appearing in the left hemifield and were paired with a horizontally lined rectangle that was either the same width or was wider, appearing in the right hemifield. For the remaining two stimulus conditions, the vertically and horizontally lined stimuli were on the right and left, respectively. The four stimulus conditions meant that three different stimuli were presented to each hemifield; a vertically lined square, a horizontally lined square, and a horizontally lined rectangle whose width was perceptually matched to the vertically lined square and was thus physically wider.

**Figure 2.**
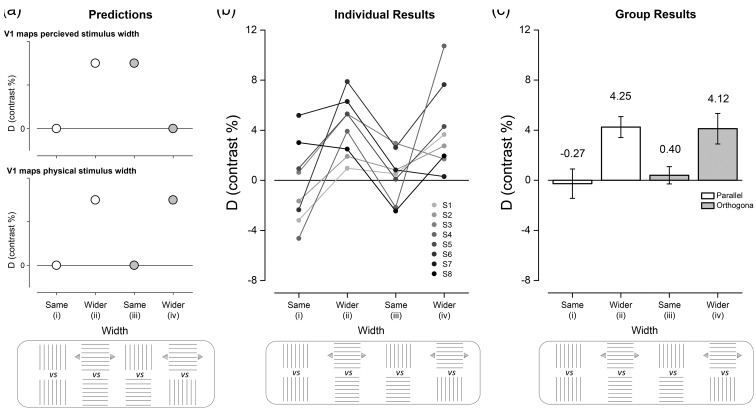
Predictions and results for BOLD responses elicited in primary visual cortex by the Helmholtz squares illusion. (a) The pattern of results based on the prediction that the extent of BOLD responses in V1 will reflect the perceived (top) or physical (bottom) width of the stimulus. The horizontal axis is labeled with the lined-stimuli that elicited the extent of BOLD responses *d*_1_ (top row) and *d*_2_ (bottom row), which were used to compute the contrast measure, Blue arrows indicate physically wider squares. (b) Individual data (circles) (c) Group mean data (bars) with error bars indicating standard error of the mean. The results fit with the prediction that V1 maps physical, not perceived width.

Our hypotheses were tested by contrasting the extent of BOLD responses elicited by these stimuli. Our outcome measure, D, to compare conditions was defined as (*d*1 − *d*2)/(*d*1 + *d*2), as shown in Figure 1d, where *d* is the distance over which the *t*-statistic exceeded 25% of its peak value for the line region of interest (ROI) along the horizontal meridian representation of V1 (Figure 1b and c). We selected 25% of the *t*-statistic as the threshold as it uniformly registered highly significant BOLD responses but also offered a consistent measure relative to the peak BOLD response in each participant. The advantage of computing D is that it is not vulnerable to cortical magnification variations between participants. Because we had two stimulus conditions (per hemifield) for which vertically lined squares were presented, our contrast measure, D, provides baseline data for the extent of BOLD responses in V1 for stimuli that were identical; we predict that D is zero (see Figure 2a i) for this baseline. The prediction for the horizontally lined square and wider, rectangular horizontally lined rectangle is that D will be positive (Figure 2a ii). This is an essential *calibration* as it allows us to determine whether we can detect changes in the extent of BOLD responses in V1 elicited by stimuli that are perceived as differing in width and which are indeed physically different in width. The magnitude of D will vary depending on whether V1 follows the illusion or the physical dimensions of the stimuli when the stimuli with differing line orientation are considered. D will either be positive (V1 maps perceived stimulus width) or zero (V1 maps the physical stimulus width) when the physically square stimuli with different line orientation are contrasted (Figure 2a iii). When the vertically lined stimulus is contrasted with the horizontally lined wider stimulus, D will either be zero (V1 maps perceived stimulus width) or positive (V1 maps the physical stimulus width; Figure 2a iv).

The difference in the physical size of the stimuli that are perceived to be of equal width is relatively small (mean 0.7°—see Table 1). It is important therefore to assess whether a shift of such a size can be detected in the retinotopic representation of V1. The well-characterized V1 cortical magnification predicts that the spread of BOLD responses due to the illusion (0.35° at each flank of the stimulus) should result in 2 mm of additional cortical activation at the inner flank of the stimulus (3° eccentricity) but only 0.5 mm at the outer flank. It is likely therefore that the resolution of fMRI will only detect shifts at representations of the near rather than the far flank (Engel, Glover, & Wandell, 1997; see Figure 1d). To achieve measuring the total ∼2.5 mm predicted change in the extent of BOLD responses, we performed fMRI at a reasonably high resolution (2.5 × 2 × 2 mm). So, while the change is small, it should be detectable in V1 with our approach. However, for other visual areas which have smaller representations of the visual field, we are unable to test our hypotheses because of the spatial resolution of our fMRI measures. It is also important to note that the differences in spatial extent that we are aiming to detect in V1 could be easily masked by relatively small eye movements. With this in mind, we had participants perform a demanding fixation task. A consequence is that this biases the BOLD responses that we will measure to stimulus-driven activity by reducing the effect of top-down feedback (Fang et al., 2008).

**Table 1.** Psychophysical Results for the fMRI Experiment.

Subject	Control condition (°)	Illusion condition (°)	Illusion strength (°)	Illusion strength (%)
S1	6.59	7.09	0.60	7.67
S2	6.56	7.38	0.82	12.5
S3	6.63	7.34	0.71	10.7
S4	6.57	7.17	0.60	9.16
S5	6.59	7.13	0.54	8.13
S6	6.62	7.79	1.17	17.8
S7	6.62	7.29	0.67	10.1
S8	6.61	7.16	0.55	8.32

*Note*. fMRI = functional magnetic resonance imaging.

### Design of the TMS Experiment

While the fMRI experiment given earlier was designed to detect stimulus-related BOLD responses in primary visual cortex and determine whether it was governed by perceptual or physical dimensions of the stimulus, the data we can derive from fMRI are correlational. Moreover, because of cortical magnification and fMRI resolution, we were restricted to detecting changes in V1 alone. We therefore designed a TMS experiment that is capable of probing the *causal* roles that V1 and two extrastriate visual areas play in the perception of the Helmholtz illusion. We designed the study as a follow-up to the fMRI study in which we detected no illusion-related BOLD responses in V1. However, one reason for this could have been the absence of a stimulus-related task. Importantly, therefore, our TMS experiment actively engaged participants in an aspect ratio judgment that highlighted the illusion. Specifically, we had participants indicate whether rectangular stimuli, which were set to be perceptually square, were taller or wider than a square. We can revisit the role of V1, therefore, by applying TMS to it during these perceptual judgments. We also reasoned that two other visual areas, LO1 and LO2, might also play a role in the Helmholtz illusion. LO1 has been shown to be orientation-selective (Larsson, Heeger, Larsson, & Heeger, 2006) and TMS to LO1 also interferes with orientation discrimination (Silson et al., 2013). Given that the stimulus difference that gives rise to the Helmholtz illusion is one of orientation alone, we asked how LO1 might be involved. LO2 offers a good control region as it lies very nearby LO1 and therefore tests the spatial specificity of any effect we might detect for LO1. Crucial to the design of this experiment is the predicted behavioral outcome that is associated with a release from the illusion. Individuals were presented with stimuli that they originally perceived to be square, but were in fact physically anisotropic, meaning a release from the illusion would result in participants’ judgments becoming *more veridical*. That is, their responses would reflect the physical anisotropy of the stimulus. We predict therefore an increase in correct judgments when TMS disrupts activity in a region of the brain that governs the illusion.

## Procedures

### Measuring the Size of the Illusion and Producing Stimuli that Null it

Key to both the experiments is the prerequisite that we can measure the Helmholtz illusion and then produce stimuli that null the illusion for each individual. For the psychophysical experiments that preceded the fMRI measurements, we presented stimuli in the left and right hemifields simultaneously for 600 ms, with the nearest edge 3° away from a central fixations cross. The lines comprising the stimuli alternated between black (300 ms) and then white (300 ms) during the 600 ms trial and were presented on a uniform gray background that was of luminance equivalent to the mean of the black and white lines. The two different phases of the stimulus were used to prevent afterimages. To create prominent illusion, each rectangular stimulus consisted of seven lines, with a duty cycle of 0.9 (thin lines on a gray background) and a spatial frequency of 0.91 cycles/degree (Mikellidou & Thompson, 2013, 2014; Thompson & Mikellidou, 2011). We spatially jittered the stimuli by up to 0.1° to prevent afterimages providing cues to physical size changes between successive trials. We used the method of constant stimuli to determine the point of subjective equality (PSE) under two different conditions.

In one condition, we presented a *reference*, 6.6° square comprising *horizontal* lines to either the left or right hemifield and in the other hemifield, we presented a *test* rectangle comprising *horizontal* lines, which could be one of the seven widths (5.7, 6.0, 6.3, 6.6, 6.9, 7.2, and 7.5°). This condition provided control data as participants should, under these stimulus conditions, have veridical perception as both the reference and test are defined by lines of the same orientation. In another condition, we presented a 6.6° *reference* square comprising *vertical* lines to either the left or right hemifield and in the other hemifield, we presented a *test* rectangle, defined by *horizontal* lines, drawn from those used in the control experiment. This condition was used to assess the size of the illusion. The height of the stimuli remained constant for all conditions. The two conditions were interleaved into single runs. Two runs were carried out, with each run comprising 700 trials (350 control and 350 illusion) with each test stimulus width presented 50 times for each condition. The participant was asked to indicate on which side (left or right) the wider of the two rectangles appeared. We derived psychometric functions for each participant and fitted them with a cumulative Gaussian. The PSE was found to be in the range of 6.56° to 6.63° for the control condition and 7.09° to 7.79° for the illusory condition. Our participants were therefore veridical in the control condition and experienced the illusion in the other condition where the horizontal stimulus width was increased between 8 and 18% in order to be perceptually matched to the vertically lined stimulus. The psychophysics took approximately 20 minutes to complete. The results for individuals in the psychophysical experiment are shown in Table 1.

The ViSaGe (Cambridge Research Systems) Visual Stimulus Generator was used, along with its MatLab (Mathworks, Natick, MA) CRS toolbox, to present calibrated stimuli on a Mitsubishi Diamond Plus 91 monitor with precision timing (viewing distance = 57 cm). The screen’s resolution was 1024 × 768 pixels and the frame rate was 60 Hz (mean luminance = 10 cdm^−2^). A CB6 (Cambridge Research Systems) response box was used to gather participants’ responses.

For the psychophysical experiment that preceded the TMS experiment, we adopted a modified approach. We reasoned that we needed to disrupt the representation of the stimulus to interfere with the illusory percept. The paired stimulus design used in fMRI would be impossible with a single TMS coil as the stimulus representation would be in both hemispheres. Even with two coils and dual TMS, the proximity of the stimulus representation in left and right V1 to the midline would not allow two coils to be separated physically to stimulate left and right V1 simultaneously. We therefore presented stimuli in only one location allowing us to stimulate its representation in V1, LO1, and LO2. We also reduced the size of the stimuli to 2.9°, so the stimulus representation in V1 was closer to the cortical surface than it would have been for the larger stimuli used in the fMRI experiment. This precaution gives us greater confidence that the distance between the TMS coil and our three targets will not vary greatly and will not therefore be the cause of any potential differences in the participant’s responses. We also changed the stimulus duration to 200 ms as this is a period over which we routinely apply TMS to disrupt performance in visual tasks and allows us to stay within published safety guidelines for TMS (Rossi et al., 2009). As a result of the changes we made to the stimuli, we needed to assess how the aspect ratios of these newly specified horizontally and vertically lined rectangles were perceived and used the following stimuli and procedure to do so.

Stimuli were black, vertically and horizontally lined, rectangles presented on a uniform gray background. As before, each rectangular stimulus consisted of seven lines, with a duty cycle of 0.9 (thin lines on a gray background), but in this case subtended 2.9° along the dimension orthogonal to the direction of the bars. The length of the bars was varied during the psychophysical experiment.

**Figure 3.**
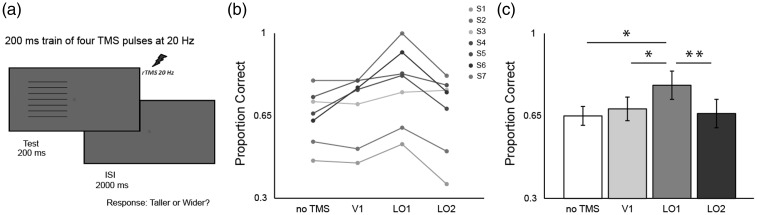
(a) Overview of experimental procedure. (b) Individual data (circles). (b) Group mean data (bars). (b) and (c) data are plotted in terms of proportion correct responses: 0.50 indicates participant guessing as expected for PSE-matched, perceptually square stimuli; 1.00 indicates correct reporting of the physically elongated stimulus dimension on all trials. Results for horizontally and vertically striped stimuli are pooled together, except in the case of S2 and S5 (see Procedure section for the TMS study examining the causal roles of V1, LO1, and LO2 in size perception). Error bars denote ± SEM. Asterisks denote a significant difference between conditions (*p < .05, **p < .01; Bonferroni-corrected).

Participants fixated a central red circle (0.15°) throughout the experiment. Stimuli were centred at 2.8° along the horizontal meridian in the visual field contralateral to planned TMS stimulation and were viewed binocularly (Figure 3a). On each trial, the absolute position of the stimulus was varied by adding a random value between 0° and ± 0.1° to both *x* and *y*. The stimulus was shown for 200 ms followed by an intertrial interval (ITI) in which the participant indicated whether the stimulus was taller or wider than a true square. To capture individual psychometric functions for both the horizontal and vertical Helmholtz stimuli, a total of six interleaved staircases (1-up, 3-down; 3-up, 1-down; 1-up, 2-down; 2-up, 1-down; two 1-up, 1-down staircases) were completed for each stimulus. Horizontally and vertically lined stimuli were randomly interleaved within the same experimental block. These procedures were used to distribute trials at informative points along the psychometric function, which was fitted using data from all trials, except the first two reversals which were removed from the analysis. The step size was initially 0.4° and halved on reversals 3–5. The staircase concluded after 14 reversals, resulting in ∼30 trials per staircase type (∼180 trials per psychometric function). A cumulative Gaussian was fitted to the data and the 0.5 point was taken at the observer’s PSE. The PSE gives the dimensions of the stimuli that were perceived as square. To account for any variance across sessions, participants completed the behavioural psychophysics three times and the average value was used in the TMS experiment. Each of the three runs took approximately 20 minutes to complete. The results for the psychophysical experiment are shown in Table 2.

**Table 2.** Psychophysical Results for the TMS Experiment.

Subject	Vertical stimulus PSE (°)	Vertical illusion strength (°)	Vertical illusion strength (%)	Horizontal stimulus PSE (°)	Horizontal illusion strength (°)	Horizontal illusion strength (%)
S1	3.41	0.51	17.59	3.47	0.57	19.66
S2	2.90	0	−0.10	3.28	0.38	12.93
S3	3.37	0.47	16.26	3.41	0.51	17.54
S4	3.23	0.33	11.39	3.27	0.37	12.89
S5	2.84	−.06	−2.22	3.49	0.59	20.24
S6	3.23	0.33	11.45	3.51	0.61	21.01
S7	3.36	0.46	15.75	3.24	0.44	11.61

*Note*. TMS = transcranial magnetic stimulation; PSE = point of subjective equality.

Stimuli were generated using Matlab and Psychtoolbox (Brainard, 1997; Kleiner et al., 2007) and displayed on a Mitsubishi Diamond Pro 2070^SB^ display (viewing distance = 57 cm) with a resolution of 1024 × 768 pixels and a refresh rate of 60 Hz.

### Retinotopic Mapping Procedures for Both fMRI and TMS Experiments

Crucial to the fMRI and TMS experiments is the need to identify retinotopic maps in each individual. For our fMRI experiment, we need to identify V1 and for the TMS experiment, we need to identify V1, LO1, and LO2. All imaging data involved in the process of retinotopic mapping were acquired on a GE 3-Tesla Signa HD Excite scanner at the YNiC, University of York.

A rotating wedge was used to map polar angle and expanding rings were used to map eccentricity and standard Fourier methods were used to analyze the retinotopic data (DeYoe et al., 1996; Engel et al., 1997; Sereno et al., 1995). Stimuli were unmasked portions of a 100% contrast radial checkerboard (14° radius) with 24 radial segments on a mean gray background. Wedges were 90° in size and rotated counterclockwise about a red fixation cross. Ring stimuli expanded about fixation. Participants maintained fixation throughout the scan. Both wedges and rings were high contrast (>98%, 400 cdm^−2^) checkerboard stimuli that reversed contrast at a rate of 6 Hz. For the fMRI experiment, four scans were collected (two wedges and two rings) and each scan contained seven cycles of wedges/rings, with 36 s per cycle. For the TMS experiments, eight scans were collected (four wedges and four rings) and each scan contained eight cycles of wedges/rings, with 36 s per cycle. For both the fMRI and TMS experiments, data were averaged across scans.

Functional data across all sessions were aligned to a canonical anatomical volume using a proton-density image acquired with the same prescription as the functional data as an intermediate alignment step. Motion correction was achieved using FSL’s MCFLIRT (Jenkinson, Bannister, Brady, & Smith, 2002) and no significant movements were seen throughout scanning. The functional time series were high-pass filtered to remove baseline drifts. We used mrVista and mrMesh analysis software to perform the retinotopic analysis and visualize data in volume and inflated cortical views (http://white.stanford.edu). Visual areas were hand drawn on these inflated cortical views according to established reversals in polar angle demarcating specific visual areas (Larsson, Heeger, et al., 2006; Wandell, Dumoulin, & Brewer, 2007).

The anatomical data that provided a canonical volume were acquired with different procedures for the fMRI study and for the follow-up TMS study. For the fMRI study, a 3D-Fast Spoiled Gradient-Recalled Echo (FSPGR) sequence was used to acquire multi-average, whole-head T1-weighted anatomical volumes for each participant (repetition time [TR] = 7.8 ms, time to echo [TE] = 3 ms, TI = 450 ms, field of view [FOV] = 290 × 290 × 176, 256 × 256 × 176 matrix, flip angle = 20°, 1.13 × 1.13 × 1.0 mm^3^). Data were obtained with an eight-channel head coil. For the TMS study, we adopted a revised protocol that we now routinely use to increase tissue contrast for automated segmentation: Three whole-head T1-weighted anatomical volumes were acquired for each subject (TR = 7.8 ms, TE = 2.7 ms, TI = 600 ms, flip angle = 12°, FOV = 256 × 256 × 176, 256 × 256 × 176 matrix, 1 × 1 × 1 mm^3^) using a 16-channel (half-head coil) and averaged. One T2*-weighted fast gradient recalled echo scan was also acquired (TR = 400 ms, TE = 4.3 ms, flip angle = 25°, field of view = 290 × 290 × 176, 256 × 256 × 88 matrix, 1.13 × 1.13 × 2 mm^3^) using a 16-channel head coil. Average T1 data were divided by the T2* data in order to correct for signal gradient resulting from the signal dropout of the 16-channel coil and to improve white/gray matter contrast. One whole-head eight-channel T1-weighted volume was acquired for each subject for use in coregistration for TMS (TR = 7.8 ms, TE = 2.9 ms, TI = 450 ms, flip angle = 20°, FOV = 290 × 290 × 176, 256 × 256 × 176 matrix, 1.13 × 1.1.13 × 1 mm^3^). For both methods of acquiring anatomical data, the average T1-weighted anatomical volume was segmented into white and gray matter for each hemisphere using Freesurfer (http://surfer.nmr.mgh.harvard.edu/). The subsequent gray-white matter segmentation was hand edited and checked for topology errors using itkGray (http://white.stanford.edu).

For the fMRI study, retinotopy data were obtained with gradient recalled echo pulse sequences to measure T2* BOLD data parallel to the calcarine sulcus (TR = 2000 ms, TE = 30 ms, FOV = 256 mm, 128 × 128 matrix, 26 contiguous slices, slice thickness = 2.5 mm, in-plane resolution = 2 × 2 mm). Magnetization was allowed to reach a steady state by discarding the first five volumes. Data were obtained with an eight-channel head coil. For the TMS study, a revised approach was adopted that exploits the increased signal to noise of a 16-channel coil and uses a slightly higher spatial resolution. Gradient recalled echo pulse sequences were used to measure BOLD signals acquired parallel to the calcarine sulcus (TR = 3000 ms, TE = 30 ms, flip angle = 90°, FOV = 192 × 192 × 78, 96 × 96 matrix, 39 contiguous slices per volume at 2 × 2 × 2 mm^3^). The first three volumes from all scans were discarded to allow the magnetization to reach magnetization steady state.

### Procedure for the fMRI Study Examining the Extent of BOLD Responses in V1 Elicited by Horizontally and Vertically Lined Stimuli

To obtain robust V1 responses in fMRI, we varied the contrast of the lined rectangles by alternating their luminance between 20 and 0 cdm^−2^ every 300 ms (contrast reversal 1.67 Hz) on a uniform grey background (10 cdm^−2^). Stimuli were rear projected onto an acrylic screen and viewed by participants lying supine in the scanner from 57 cm via a front-silvered mirror mounted onto the MRI head coil. To determine whether regions of the early visual cortex have a representation for perceptual differences, we presented stimuli on either side of fixation while acquiring T2* weighted volumes.

In the first out of four stimulus conditions employed, Helmholtz’s squares consisting of a horizontally striped and a vertically striped square of identical dimensions (6.6 × 6.6°; seven lines; duty cycle = 0.9; spatial frequency = 1.30c/°). The vertically and horizontally lined squares appeared in the left and right hemifields, respectively, in one condition and vice versa in the other. In the other two conditions, in order to compensate for the Helmholtz’s squares illusion, the pre-fMRI psychophysics results from each individual were used to adjust the width of the horizontally striped square such that it would be perceptually matched with the vertically lined square. As before, the vertically and horizontally lined stimuli appeared in left and right hemifields, respectively, in one condition, and vice versa in another. Each stimulus condition was presented for a block of 9 s followed by a 9 s block of uniform gray. In total, eight blocks per condition were presented in a pseudorandom order. The duration of the run was therefore 4 conditions × 8 blocks × 18 s = 576 s. Each participant completed two runs. Because the block length of 9 s was much longer than the 0.6 s stimulus duration we used in psychophysics, we tested one of our participants with this longer (9 s) stimulus duration and found that their results were unchanged (control PSE: 6.57° [short duration 0.6 s] vs. 6.59° [long duration 9 s]; illusion PSE: 7.17° [short duration 0.6 s] vs. 7.20° [long duration 9 s]).

We used a demanding central attention task to ensure that participants maintained fixation, as small shifts in gaze could hinder our ability to detect small changes in the extent of cortical BOLD responses. A fixation task also serves to minimize top-down influences on V1. In the task, participants fixate a small white central square and count the number of times the position of a smaller red square, which was flashed randomly at one of the eight immediately surrounding locations, appeared at 12 o’clock. At the end of each run, participants reported the count. The accuracy on this very demanding task varied between 70 and 97%.

The fMRI acquisition parameters were identical to those used for the retinotopic mapping data and yielded the relatively high in-plane resolution of 2 × 2 mm^2^ that was required to test our hypotheses. The fMRI data were also motion corrected and aligned to the canonical anatomical data as specified earlier. We then computed *t*-statistics from general linear modelling as implemented in mrVista. In this process, data were first high pass filtered with a boxcar function of duration of 18 s. Otherwise, data were left spatially unsmoothed but were up-sampled when transformed to the anatomical space. The spatial distribution of the *t*-statistic data along the representation of the horizontal meridian was analyzed as specified in the design of the study (described earlier and see also Figure 1).

### Procedure for the TMS Study Examining the Causal Roles of V1, LO1, and LO2 in Size Perception

For the TMS study, we assessed our retinotopic mapping data to locate the targets for stimulation. The identification of LO1 and LO2 is identical to that described previously (Silson et al., 2013). V1 was identified from the polar angle data as the hemifield map found in calcarine cortex. Targets for TMS were selected from the hemisphere in which LO1 and LO2 were most clearly identifiable, resulting in the left hemisphere being stimulated in five observers (Table 3). For each participant, centroids for V1, LO1, and LO2 were calculated for accurate TMS coil targeting using the Brainsight system (Rogue Research). The distance between these centroids is shown in Table 3. Further, we included an optimal trajectory for all ROIs that specified the angular approach of the TMS coil to the target region. For LO1 and LO2, the trajectories were set approximately parallel to one another, each going through the center of mass the respective ROI. Using retinotopic eccentricity maps, we restricted the V1 ROI to the center of the Helmholtz stimuli (2.8°). To maximize the effects of TMS along the horizontal meridian, a trajectory was created in Brainsight that aligned the TMS coil down the calcarine sulcus through the center of this ROI. Having gathered fMRI data for relatively large, more eccentric Helmholtz stimuli, we knew that we needed to create smaller, less eccentric Helmholtz stimuli in order to make them accessible to TMS stimulation.

**Table 3.** V1, LO1, and LO2 Centroids.

Subject	Hemisphere	V1			LO1			LO2			V1:LO1 (mm)	LO1:LO2 (mm)
		X	Y	Z	X	Y	Z	X	Y	Z		
S1	Left	−9.83	−93.55	−8.94	−25.25	−90.16	8.99	−35.75	−81.29	6.19	23.87	15.68
S2	Left	−4.10	−91.25	−7.64	−20.58	−89.95	8.26	−28.86	−87.44	3.25	22.11	10.15
S3	Right	7.22	−90.45	−4.86	20.70	−92.00	1.76	26.82	−87.85	−0.31	15.39	7.28
S4	Left	−10.535	−94.106	−17.101	−19.67	−95.90	−6.79	−28.50	−90.17	−2.84	19.22	7.41
S5	Right	11.51	−93.35	−0.59	30.32	−82.99	5.81	36.93	−80.15	3.05	22.21	7.86
S6	Left	−7.90	−92.06	−7.23	−34.268	−78.671	5.761	−36.66	−76.85	2.84	30.71	8.38
S7	Left	−5.97	−88.73	−11.50	−23.83	−84.93	1.65	−34.10	−77.77	2.26	23.08	13.05

*Note*. Talairach centroids along with the actual distance between V1-LO1 centroids and LO1-LO2 centroids are given for all seven participants.

For each 200 ms stimulus presentation (Figure 3a), a train of four biphasic pulses, separated by 50 ms (20 Hz) were delivered at 70% of the max stimulator output (2.6 T). As we did not have specific predictions about feedforward versus feedback processing, we used this protocol to cover time periods that should be influenced by both processes. Pulses were delivered by a Magstim ‘figure of 8’ coil connected to a Magstim Rapid 2 stimulator. Participants were seated in a custom built chair with chin rest and temple support. The coil was mechanically secured, placed directly above the cortical target, and pressed flush against the participants scalp to minimize the coil-target distance. The coil was tracked in real time to provide trial-by-trial measurements of coil-target distance and coil-target offset.

Each subject underwent four counterbalanced sessions (no TMS and TMS to V1, LO1, or LO2). During each session, four trial types were presented: Horizontally lined stimuli that were (a) perceptually and (b) physically square, and vertically lined stimuli that were (a) perceptually and (b) physically square. There were 50 presentations of each trial type (total 200 trials). The no TMS session took approximately 10 minutes to complete and each TMS session took approximately 20 minutes to complete. The order of trials was randomized with a minimum ITI of 2000 ms in addition to the participants’ reaction time. Trials for which coil-target displacement was large (>2.5 mm), or reaction time was greater than 2000 ms after the offset of the stimulus, were removed from the analysis (∼1%). We included trials in which physically square stimuli were presented because they can allow us to test whether participant response become biased to *wider* or *taller* as a result of TMS or as a result of the orientation of the stimuli. For example, the predicted release from the illusion would mean that horizontally lined stimulus that was set to be perceptually square would be perceived as wider, a response that might arise if a bias related to the stimulus orientation occurs. However, such a bias would be expressed for the physically square, horizontally lined stimuli, so examination of responses to the square stimuli will show whether an orientation and TMS-dependent bias in aspect ratio judgments arises.

Two observers (S2 and S5) did not experience the Helmholtz illusion for the vertically striped stimuli (see Table 2). We would not expect to be able to elicit a release from the illusion when the illusion is not present and therefore excluded trials to the vertically lined stimuli for these two observers from the analysis. We recruited eight participants for the TMS experiment. One participant did not complete the experiment due to salient phosphenes which they found distracted them from the task.

## Results

### Assessing the Extent of BOLD Responses in V1 and Its Relationship With an Illusory Percept of Size

For each observer, we first established psychophysically the extent of the Helmholtz squares illusion (see Experimental Procedures, Table 1). By increasing the width of the horizontally lined squares, we generated stimuli that cancelled the illusion; these stimuli were perceptually square, but physically rectangular (Figure 1a). During the fMRI experiments, participants viewed three different stimuli: a vertically lined square, a horizontally lined square, and a horizontally lined rectangle whose width was perceptually matched to the vertically lined square, but as a result was physically wider. These stimuli were presented in a different pairing (see Methods section) that allowed the calculation of contrasts to examine whether V1 maps the perceptual or physical dimensions on the stimuli. Figure 2a illustrates these contrasts and the associated predictions for V1 mapping the perceived stimulus width (top panel) or V1 mapping the physical stimulus width (bottom panel).

Comparisons (i) and (ii) allow us to gauge whether our measures are sensitive to detect changes in physical size which are equivalent to the perceived changes observers experience in the illusion (Figure 2a). Comparisons (iii) and (iv) will have very different results depending on the role of V1 in the Helmholtz squares illusion (Figure 2a). If stimulus-driven BOLD responses in V1 underlies the illusion, stimuli of matched physical width defined by orthogonal line orientation will elicit different activity profiles, whereas those matched for perceived size should register no difference in activity profile (Figure 2a—top row). If however stimulus-driven BOLD responses follows the physical dimensions of the stimulus, we predict the opposite (Figure 2a—bottom row).

Data showing D for each individual are shown in Figure 2b. The pattern of results is largely uniform across participants. This pattern is confirmed in the group means shown in Figure 2c. The value of D is distributed around zero for stimuli of the same orientation and dimensions (contrast (i)), whereas the contrast between stimuli of the same orientation, but different dimensions (contrast (ii)) is positive. Taken together, these results confirm that our scanning parameters appeared sensitive enough to detect relatively small physical differences in V1. The crucial comparisons to determine whether V1 maps the perceived size of the stimulus come from contrasts iii and iv. For contrast iii, D is again distributed around zero showing that for stimuli of different orientation, but identical physical dimensions, the extent of BOLD responses in V1 is very similar. In the situation when the stimuli differ in orientation, but are matched perceptually, D is positive reflecting V1’s sensitivity to unequal size of the stimuli. Qualitatively, therefore, the pattern of the data (Figure 2b) is indistinguishable from the lower panel of Figure 2a in which the prediction of the results for “V1 maps physical stimulus width” is given.

The data fit neatly in a framework that can be assessed statistically with a two-way analysis of variance (ANOVA), the factors being physical width (same/wider) and orientation (parallel/orthogonal). A significant main effect of physical width, *F*(1, 7) = 11.4, *p = .*012), but not orientation, *F*(1, 7) = 0.05, *p = .*826, was found. The interaction between physical width and orientation was not significant, *F*(1, 7) = 0.33, *p = .*582. The extent of BOLD responses along the representation of the horizontal meridian is therefore entirely explained by the physical dimensions of the stimuli. It is important to note that this is not a null result; the extent of BOLD responses *changes significantly* in V1 as a result of a change in the dimension of the stimulus, but the change is not dependent on the orientation of the stimuli, which does affect our perception.

### The Causal Role of V1, LO1, and LO2 in an Illusory Percept of Size

Having established that stimulus-driven BOLD responses in V1 do not underpin the Helmholtz squares illusion, we asked whether this result arose because the illusion is underpinned by neural processing outside of V1. To answer this question, we had participants make judgments on perceptually square horizontally and vertically lined stimuli while we applied TMS to three candidate regions: V1 and extrastriate LO1 and LO2 (Figure 3a; Larsson, Heeger, et al., 2006). As before, each participant was tested ahead of the TMS session to determine the dimensions of the rectangle that were perceived square (Table 2).

If TMS successfully disrupts neural processing specific to one’s percept of the Helmholtz squares illusion, a release from the illusion would be experienced. The more the illusion dissipates, the more the physical anisotropy of the stimuli will become apparent, and therefore the more correct responses will be made by the observer. If TMS does not interfere with processing specific to the illusion, we would expect no change in the proportion of correct responses. That is we expect responses around chance (0.5) for a stimulus that is perceptually square where the participant has to make a forced choice response—taller or wider? For the no TMS condition, responses were not at floor (0.5) but around 0.65 (Figure 3c), suggesting that during the psychophysics, participants had a tendency to overcorrect for the Illusion. Even at 0.65, there is plenty of room for a release from the illusion (toward 1.0), so this overcorrection did not hinder the design of the study.

The individual data detailing the proportion of correct responses across the baseline no TMS and TMS (V1, LO1, and LO2) sessions are shown in Figure 3b. It is clear that in each observer (except S3), TMS to LO1 results in the greatest number of veridical responses. This is reflected in the group means as shown in Figure 3c. A one-way repeated measures ANOVA revealed a significant main effect of TMS site stimulation, *F*(3, 18) = 9.93, *p* = .00004. We compared proportion of correct responses among the four conditions by conducting six pairwise *t*-tests (two-tailed, Bonferroni corrected). Compared to the no TMS condition (*M* = 0.65*, SD* = .12), TMS of area V1 does not compromise the strength of the illusion, *M* = 0.68*, SD* = .14; *t*(6) = 1.49, *p = .*99. Interestingly, only disruption of area LO1 compromised significantly the strength of the illusion compared with the no TMS condition, *M* = 0.78*, SD* = .17; *t*(6) = 3.85, *p = .*048, emphasizing the importance of LO1 in processing of orientation information (Larsson, Landy, & Heeger, 2006; Silson et al., 2013; Tibber, Anderson, Melmoth, Rees, & Morgan, 2009). Disruption of LO1 processing lead to significantly more veridical responses compared with disruption of V1, *t*(6) = 4.14, *p = .*036. Further, disruption of LO1 led to significantly more veridical responses compared with disruption of LO2, *t*(6) = 4.21, *p = .*03. Selective stimulation of LO2 (*M* = 0.66*, SD* = .17) did not significantly affect the proportion of veridical responses compared with no TMS, *t*(6) = 0.64, *p = *.99, and V1 stimulation, *t*(6) = 1.0, *p = .*99).

### Exploring Potential Explanations of the TMS Results

The TMS task reported here is largely robust to nontask specific effects such as increased distraction owing to discomfort during TMS. Task distraction would most likely result in one guessing, causing responses to move toward floor (0.5) in the opposite direction to a predicted release from the illusion. Still, we wanted to rule out whether TMS to LO1 or LO2 interfered with visual tasks in general. That is, was the TMS site-specific effect we observed for the aspect ratio judgments also specific to that task? To do this, we had six of our seven participants perform a contour integration task (not part of the original design reported here). Participants were required to indicate in which of two temporal intervals, a contour, defined by aligned Gabor elements within an array of randomly orientated Gabors, appeared (Field, Hayes, & Hess, 1993). We found no effect of the site of TMS on performance in this task, *F*(2, 10) = 0.78, *p* = .93. Task performance for TMS to LO1 and LO2 was, *M* = 0.72 *SD* = .02 and *M* = 0.73 *SD* = .05, respectively. A two (Experiment: contour integration, Helmholtz) by three (ROI: no TMS, LO1, and LO2) repeated measures ANOVA found no main effect of experiment, *F*(1,5) = 0.22, *p* = .656, a significant main effect of ROI *F*(2, 10) = 4.14, *p* = .049, and importantly, a significant interaction between experiment and ROI, *F*(2, 10) = 8.72, *p* = .006), indicating that the effect of TMS to LO1 was specific to the Helmholtz stimuli reported here.

Because response bias may occur during TMS stimulation, we designed the experiment to include stimuli to explore this possibility (see Methods section). A response bias related to the orientation of the stimuli could provide a pattern of results that is interpreted as a release from the illusion (a bias in reporting wider for horizontal stimuli and taller for vertical stimuli). To check for response bias, we presented physically square stimuli of different line orientation which normally show the illusion. A one-way ANOVA for the horizontally lined, *F*(3, 18) = 0.29, *p* = .831, and vertically lined, *F*(3, 18) = 1.03, *p* = .401, physically square stimuli showed responses to these stimuli to be constant across all experimental conditions (no TMS, V1, LO1, and LO2) ruling out the possible role of observer response bias in this experiment.

**Figure 4.**
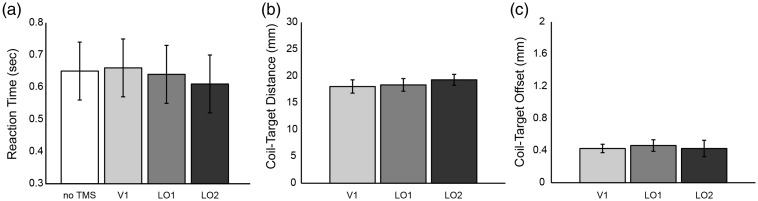
Plots of potentially confounding variables for the PSE-matched Helmholtz stimuli. (a) Reaction time. (b) Coil-target distance. (c) Coil-target offset. Error bars represent the standard error of the mean. No significant differences were found between any of the conditions reported here.

A speed-accuracy trade-off, although unlikely, could cause the pattern of results reported here. That is, participants may take longer to respond in order to increase accuracy. Reaction times across all conditions are shown in Figure 4a. No significant differences were found, *F*(3, 18) = .338, *p* = .798, ruling out an explanation based on a speed–accuracy trade-off.

A change in coil-target distance or coil-target error (the offset between the desired and actual position of the TMS coil) could also account for the pattern of results reported here. No differences were found between conditions for these measures: Figure 4b coil-target distance, *F*(2, 12) = .251, *p* = .782; Figure 4c coil-target offset, *F*(2, 12) = .039, *p* = .962. These results suggest that neither changes in coil-target distance nor coil-target error can account for the pattern of results reported here.

Importantly, as we discussed earlier, the effective depth of the figure-8 coil used in this experiment is 2 to 3 cm (Deng, Lisanby, & Peterchev, 2013; Thielscher & Kammer, 2004). Figure 4b shows that the average coil-target distance for V1, LO1, and LO2, was 1.80 cm, 1.83 cm, and 1.93 cm, respectively, indicating that TMS is effective and equally so across our stimulation sites. Further, the coil-target offset was very small<0.5 mm, indicating accurate TMS coil positioning across conditions (Figure 4c).

## Discussion

We established psychophysically the extent of the illusion and how to generate stimuli that cancelled it; these stimuli were perceptually square, but physically rectangular. Crucially, we could use these stimuli to probe whether visual cortex mapped physical or perceptual size. First, we confirmed that the extent of BOLD responses in V1 followed the physical dimensions of the stimulus rather than its perceived dimensions. These results therefore served to confirm that the processing specific to V1 was not sufficient to give rise to the illusion (Fang et al., 2008). Second, during perceptual judgments of stimuli that cancelled the illusion, we found that TMS to LO1, and not V1 or LO2, shifted participants’ perception from the illusory to the physical dimensions of the stimuli.

While BOLD responses in V1 can undoubtedly be modulated by illusory percepts (Fang et al., 2008; He et al., 2015; Kok et al., 2016; Kok & De Lange, 2014; Murray et al., 2006; Pooresmaeili et al., 2013; Sperandio et al., 2012), our results show that V1 does not have the capacity to cause the illusory percept itself. We are able to draw this conclusion on the basis that we were able to document small, but significant changes in the extent of BOLD responses in primary visual cortex that followed only physical, rather than perceptual dimensions of the stimulus. Had primary visual cortex followed the illusion, our methods were sensitive enough to detect it. Importantly, and in contrast with previous reports, we provide causal evidence illustrating that extrastriate, rather than primary visual cortex, is responsible for the experience of a size illusion. This result stands alongside existing evidence suggesting other illusions may have a neural locus in the extrastriate lateral occipital area as observed for the Müller-Lyer illusion (Weidner & Fink, 2007) and Kanizsa-type illusory figures (Wokke, Vandenbroucke, Scholte, & Lamme, 2012).

We designed our study after Fang et al. (2008) who found illusion-related BOLD responses in V1 dissipated when attention was occupied using a demanding central fixation task. The authors reasoned that reducing attention to the 2-D contextual surround that gives rise to the Ponzo illusion likely reduces feedback to V1 from extrastriate regions known to process these 2-D depth cues. By similarly occupying attention in the fMRI study reported here, we aimed to reduce any effects of top-down feedback and thus bias our measures to look at stimulus-driven BOLD responses in V1. However, more recent work published since we designed our study suggests that occupying attention may not rule out feedback signals to V1. Kok et al. (2016) examined perceptual completion of Kanizsa-type figures and found illusion-related BOLD responses in the in the deep layer of V1, known to receive extrastriate feedback, when attention was occupied centrally. It should be noted that although no significant difference was observed, there was a reduction in illusion-related BOLD responses when attention was occupied using a more demanding task (Kok & De Lange, 2014). Importantly then, by simply manipulating attention, we cannot distinguish between V1-specific and feedback accounts of illusion-related BOLD responses in V1, as in both cases discussed earlier, the illusion-related BOLD responses in V1 can be attributed to feedback (Fang et al., 2008; Kok & De Lange, 2014). Thus, it is important to examine what causal roles visual areas play in illusions with the use of TMS. The efficacy of such an approach has been demonstrated by Maus et al. who find only TMS to MT+, but not V1, reduces the flash-lag illusion (Maus, Ward, Nijhawan, & Whitney, 2013). These TMS findings dovetail with evidence for a neural correlate of the flash-lag effect in MT+, and not V1, observed when participants’ attention was occupied centrally (Maus, Fischer, & Whitney, 2013). During the TMS study reported here, we had participants fixate centrally while assessing the aspect ratio of the stimuli presented to them, rather than perform the demanding fixation task we used in fMRI. The absence of an effect of TMS to V1 during a stimulus-related task means that even if V1 were to show illusion-related BOLD responses, which we may have failed to detect because of the absence of a stimulus-related task, it does not appear to play a causal role in the illusory percept. It is important to note that there is no methodological reason why we should have encountered a lack of effect of TMS to V1. We ensured that the stimuli were located such that their representation in V1 was relatively near the scalp surface, which meant that the distance between the coil and neural tissue was not significantly different across our three stimulation sites (V1, LO1, and LO2; Figure 4). The capacity for TMS to disrupt neural processing should therefore be equal for all sites.

While it is plausible that many reports of illusory effects correlating with V1 BOLD responses reflect feedback from higher visual areas (Fang et al., 2008; Kok et al., 2016; Kok & De Lange, 2014; Murray et al., 2006), three recent reports may prove to be exceptions. First, changes in perceived size, resulting from adaptation, could originate in V1. However, similar effects found in area V2 (Pooresmaeili et al., 2013) pose a challenge in distinguishing between feedforward and feedback propagation of signals (Chouinard & Ivanowich, 2014). Second, while there is compelling evidence demonstrating that Emmert’s illusion can elicit a commensurate distortion of V1 retinotopy (Sperandio et al., 2012), V1 itself is not essential to experience this illusory percept. This is described in the case of patient D.B. whom, having had their right primary visual cortex removed, still experienced Emmert’s illusion as a *prime-sight*’ in their left visual field (Weiskrantz, Cowey, & Hodinott-Hill, 2002). Third, distortions to Gabor stimuli found in Macaque V1 are predicted based on the receptive field properties of V1 neurons, a finding that is mirrored in human psychophysical data (Michel et al., 2013). It is likely however that this effect is specific to stimuli that matched the dimensions of the putative receptive fields. Our results show that at a different scale, for the Helmholtz stimuli used here, the opposite perceptual distortion is found and that this distortion is not reflected in V1 BOLD responses.

So, it appears that while evidence exists for a change in the BOLD response profile of V1 that follows visual illusions (He et al., 2015; Kok et al., 2016; Kok & De Lange, 2014; Murray et al., 2006; Ni, Murray, & Horwitz, 2014; Sperandio et al., 2012), recent work suggests it likely originates from feedback from extrastriate cortex (Fang et al., 2008; Kok et al., 2016; Kok & De Lange, 2014; Weiskrantz et al., 2002) and may not play a causal role in our perception. However, to make such conclusions requires careful examination of the neurochronometric processing between V1 and extrastriate regions. Our approach to this was to select a TMS protocol that covered the canonical time periods for both feedforward and feedback processing between V1 and extrastriate regions (see Methods section). By choosing this protocol, we believe that we captured both feedforward and feedback processing related to the Helmholtz illusion during TMS stimulation. Alternative, elegant strategies have also been used; Wokke et al. show using single and double-pulse TMS that one’s percept of Kanizsa-type illusory figures is disrupted at an early, feedforward time signature in LO, and a late, feedback time signature in V1, suggesting that both early processing in LO and feedback to V1 contribute to the perceptual completion of these figures (Wokke et al., 2012). It is important then that future studies also examine such feedforward and feedback processing by using TMS pulses of varying latencies. This neurochronometric approach could be used to study different illusions, including those that require 2-D contextual surrounds (e.g., Ponzo), to explore whether V1 is causally involved in the illusion or if specific extrastriate loci underpin those illusions (Maus, Fischer, et al., 2013; Wokke et al., 2012). Further, future studies could examine potential roles of extrastriate regions other than LO1 and LO2 in the Helmholtz illusion.

One question is how TMS stimulation to orientation-specific LO1 may affect a release from the illusion, whereas TMS stimulation to shape-specific LO2 does not (Silson et al., 2013). Given that the Helmholtz illusion is dependent on orientation, disrupting orientation-specific processing may be expected to result in a release from the illusion as we observed with stimulation to LO1—in a classic psychophysical sense, we can think of perception and thus the PSE shifting toward the veridical dimension of the physically stretched stimulus. Disrupting shape-specific processing as would be predicted when stimulating LO2 should lead only to shape estimates becoming more variable, affecting the just noticeable difference without shifting the PSE. Further research could ask whether such effects do result from TMS to LO2, but it is noted that TMS experiments of this type would require a very large number of trials to fully capture the psychometric function. Due to TMS safety, concerns (Rossi et al., 2009), and the four stimulus types used here, we used a limited number of trials and did therefore not capture full psychometric functions. The influence of TMS on neural processing is not entirely clear. However, the disruption of orientation-specific processing in LO1 is likely a result of increased noise, which degrades an orientation signal that gives rise to the illusion.

To conclude, we identify a specific extrastriate area, LO1, that underpins an illusion of size dependent upon the orientation of lined rectangles. Moreover, V1 appears not to be causally involved in the illusory percept, a finding that may also generalize to other illusions of size.

## References

[bibr1-2041669516667628] Blakemore C. Garner E. T. Sweet J. A. (1972) The site of size constancy. Perception 1: 111–119. doi:10.1068/p010111. 4219227 10.1068/p010111

[bibr2-2041669516667628] Brainard D. H. (1997) The psychophysics toolbox. Spatial Vision 10: 433–436. 9176952

[bibr3-2041669516667628] Chouinard P. A. Ivanowich M. (2014) Is the primary visual cortex a center stage for the visual phenomenology of object size? The Journal of Neuroscience: The Official Journal of the Society for Neuroscience 34: 2013–2014. doi:10.1523/JNEUROSCI.4902-13.2014. 24501343 10.1523/JNEUROSCI.4902-13.2014 PMC6608541

[bibr4-2041669516667628] Deng Z. De Lisanby S. H. Peterchev A. V. (2013) Electric field depth-focality tradeoff in transcranial magnetic stimulation: Simulation comparison of 50 coil designs. Brain Stimulation 6: 1–13. doi:10.1016/j.brs.2012.02.005. 22483681 10.1016/j.brs.2012.02.005 PMC3568257

[bibr5-2041669516667628] DeYoe E. A. Carman G. J. Bandettini P. Glickman S. Wieser J. Cox R. Neitz J. (1996) Mapping striate and extrastriate visual areas in human cerebral cortex. Proceedings of the National Academy of Sciences of the United States of America 93: 2382–2386. doi:10.1073/pnas.93.6.2382. 8637882 10.1073/pnas.93.6.2382 PMC39805

[bibr6-2041669516667628] Engel S. A. Glover G. H. Wandell B. A. (1997) Retinotopic organization in human visual cortex and the spatial precision of functional MRI. Cerebral Cortex (New York, N.Y.: 1991), 7, 181–192. Retrieved from http://www.ncbi.nlm.nih.gov/pubmed/9087826. 10.1093/cercor/7.2.181 9087826

[bibr7-2041669516667628] Fang F. Boyaci H. Kersten D. Murray S. O. (2008) Attention-dependent representation of a size illusion in human V1. Current Biology: CB 18: 1707–1712. doi:10.1016/j.cub.2008.09.025. 18993076 10.1016/j.cub.2008.09.025 PMC2638992

[bibr8-2041669516667628] Field D. Hayes A. Hess R. (1993) Contour integration by the human visual system: Evidence for a local “association field.”. Vision Research 33: 173–193. Retrieved from http://www.sciencedirect.com/science/article/pii/004269899390156Q. 8447091 10.1016/0042-6989(93)90156-q

[bibr9-2041669516667628] He D. Mo C. Wang Y. Fang F. (2015) Position shifts of fMRI-based population receptive fields in human visual cortex induced by Ponzo illusion. Experimental Brain Research 233: 3535–3541. doi:10.1007/s00221-015-4425-3. 26314755 10.1007/s00221-015-4425-3

[bibr10-2041669516667628] Helmholtz, H., & Southall, J. (1925). *Treatise on physiological optics. Volume III. The perceptions of vision* (Trans., J. P. C. Southhall). New York, NY: Optical Society of America.

[bibr11-2041669516667628] Jenkinson M. Bannister P. Brady M. Smith S. (2002) Improved optimization for the robust and accurate linear registration and motion correction of brain images. NeuroImage 17: 825–841. doi:10.1016/S1053-8119(02)91132-8. 12377157 10.1016/s1053-8119(02)91132-8

[bibr12-2041669516667628] Kleiner M. Brainard D. H. Pelli D. Ingling A. Murray R. Broussard C. (2007) What’s new in Psychtoolbox-3? Perception 36: 1.

[bibr13-2041669516667628] Kok P. Bains L. J. van Mourik T. Norris D. G. de Lange F. P. (2016) Selective activation of the deep layers of the human primary visual cortex by top-down feedback. Current Biology 26: 371–376. doi:10.1016/j.cub.2015.12.038. 26832438 10.1016/j.cub.2015.12.038

[bibr14-2041669516667628] Kok P. De Lange F. P. (2014) Shape perception simultaneously up- and downregulates neural activity in the primary visual cortex. Current Biology 24: 1531–1535. doi:10.1016/j.cub.2014.05.042. 24980501 10.1016/j.cub.2014.05.042

[bibr15-2041669516667628] Larsson J. Heeger D. J. Larsson J. Heeger D. (2006) Two Retinotopic Visual Areas in Human Lateral Occipital Cortex. Journal of Neuroscience 26: 1–15. 10.1523/JNEUROSCI.1657-06.2006 PMC1904390 17182764

[bibr16-2041669516667628] Larsson J. Landy M. S. Heeger D. J. (2006) Orientation-selective adaptation to first- and second-order patterns in human visual cortex. Journal of Neurophysiology 95: 862–881. doi:10.1152/jn.00668.2005. 16221748 10.1152/jn.00668.2005 PMC1538978

[bibr17-2041669516667628] Maus G. W. Fischer J. Whitney D. (2013) Motion-dependent representation of space in area MT+. Neuron 78: 554–562. doi:10.1016/j.neuron.2013.03.010. 23664618 10.1016/j.neuron.2013.03.010 PMC3654409

[bibr18-2041669516667628] Maus G. W. Ward J. Nijhawan R. Whitney D. (2013) The perceived position of moving objects: Transcranial magnetic stimulation of area MT+ reduces the flash-lag effect. Cerebral Cortex 23: 241–247. doi:10.1093/cercor/bhs021. 22302116 10.1093/cercor/bhs021 PMC3513962

[bibr19-2041669516667628] Michel M. M. Chen Y. Geisler W. S. Seidemann E. (2013) An illusion predicted by V1 population activity implicates cortical topography in shape perception. Nature Neuroscience 16: 1477–1483. doi:10.1038/nn.3517. 24036915 10.1038/nn.3517 PMC3889209

[bibr20-2041669516667628] Mikellidou K. Thompson P. (2013) The vertical-horizontal illusion: Assessing the contributions of anisotropy, abutting, and crossing to the misperception of simple line stimuli. Journal of Vision 13: 1–11. doi:10.1167/13.8.7. 10.1167/13.8.7 23838607

[bibr21-2041669516667628] Mikellidou K. Thompson P. (2014) Crossing the line: Estimations of line length in the Oppel- Kundt illusion Kyriaki Mikellidou. Jorunal of Vision 14: 1–10. doi:10.1167/14.8.20. 10.1167/14.8.20 25057945

[bibr22-2041669516667628] Murray S. O. Boyaci H. Kersten D. (2006) The representation of perceived angular size in human primary visual cortex. Nature Neuroscience 9: 429–434. doi:10.1038/nn1641. 16462737 10.1038/nn1641

[bibr23-2041669516667628] Ni A. M. Murray S. O. Horwitz G. D. (2014) Object-centered shifts of receptive field positions in Monkey primary visual cortex. Current Biology 24: 1653–1658. doi:10.1016/j.cub.2014.06.003. 25017208 10.1016/j.cub.2014.06.003 PMC4123419

[bibr24-2041669516667628] Pooresmaeili A. Arrighi R. Biagi L. Morrone M. C. (2013) Blood oxygen level-dependent activation of the primary visual cortex predicts size adaptation illusion. The Journal of Neuroscience: The Official Journal of the Society for Neuroscience 33: 15999–16008. doi:10.1523/JNEUROSCI.1770-13.2013. 24089504 10.1523/JNEUROSCI.1770-13.2013 PMC4888977

[bibr25-2041669516667628] Sereno M. I. Dale A. M. Reppas J. B. Kwong K. K. Belliveau J. W. Brady T. J. Tootell R. B. (1995) Borders of multiple visual areas in humans revealed by functional magnetic resonance imaging. Science (New York, N.Y.) 268: 889–893. doi:10.1126/science.7754376. 10.1126/science.7754376 7754376

[bibr26-2041669516667628] Silson E. H. McKeefry D. J. Rodgers J. Gouws A. D. Hymers M. Morland A. B. (2013) Specialized and independent processing of orientation and shape in visual field maps LO1 and LO2. Nature Neuroscience 16: 267–269. doi:10.1038/nn.3327. 23377127 10.1038/nn.3327

[bibr27-2041669516667628] Sperandio I. Chouinard P. A. Goodale M. A. (2012) Retinotopic activity in V1 reflects the perceived and not the retinal size of an afterimage. Nature Neuroscience 15: 540–542. doi:10.1038/nn.3069. 22406550 10.1038/nn.3069

[bibr28-2041669516667628] Sterzer P. Rees G. (2006) Perceived size matters. Nature Neuroscience 440: 91–95. doi:10.1038/nature04262. 10.1038/nn0306-302b 16498422

[bibr29-2041669516667628] Thielscher A. Kammer T. (2004) Electric field properties of two commercial figure-8 coils in TMS: calculation of focality and efficiency. Clinical Neurophysiology: Official Journal of the International Federation of Clinical Neurophysiology 115: 1697–1708. doi:10.1016/j.clinph.2004.02.019. 15203072 10.1016/j.clinph.2004.02.019

[bibr30-2041669516667628] Thompson P. Mikellidou K. (2011) Applying the Helmholtz illusion to fashion: Horizontal stripes won’t make you look fatter. I-Perception 2: 69–76. doi:10.1068/i0405. 23145226 10.1068/i0405 PMC3485773

[bibr31-2041669516667628] Tibber M. S. Anderson E. J. Melmoth D. R. Rees G. Morgan M. J. (2009) Common cortical loci are activated during visuospatial interpolation and orientation discrimination judgements. PLoS One 4: e4585, doi:10.1371/journal.pone.0004585. 19238207 10.1371/journal.pone.0004585 PMC2642631

[bibr32-2041669516667628] Wandell B. A. Dumoulin S. O. Brewer A. A. (2007) Visual field maps in human cortex. Neuron 56: 366–383. doi:10.1016/j.neuron.2007.10.012. 17964252 10.1016/j.neuron.2007.10.012

[bibr33-2041669516667628] Weidner R. Fink G. R. (2007) The neural mechanisms underlying the Muller-Lyer illusion and its interaction with visuospatial judgments. Cerebral Cortex 17: 878–884. doi:10.1093/cercor/bhk042. 16707733 10.1093/cercor/bhk042

[bibr34-2041669516667628] Weiskrantz L. Cowey A. Hodinott-Hill I. (2002) Prime-sight in a blindsight subject. Nature Neuroscience 5: 101–102. doi:10.1038/nn793. 11788836 10.1038/nn793

[bibr35-2041669516667628] Wokke M. E. Vandenbroucke A. R. E. Scholte H. S. Lamme V. A. F. (2012) Confuse your illusion: Feedback to early visual cortex contributes to perceptual completion. Psychological Science 24: 63–71. doi:10.1177/0956797612449175. 23228938 10.1177/0956797612449175

